# Optimization of Enzymolysis Modification Conditions of Dietary Fiber from Bayberry Pomace and Its Structural Characteristics and Physicochemical and Functional Properties

**DOI:** 10.3390/molecules29143415

**Published:** 2024-07-21

**Authors:** Zhaolin Zhang, Qin Ruan, Xiaoming Sun, Jianfeng Yuan

**Affiliations:** 1 Xingzhi College, Zhejiang Normal University, Lanxi 321100, China; zhangzhaolin@zjnu.edu.cn (Z.Z.); ruanqin@zjnu.cn (Q.R.); sunxm64@zjnu.cn (X.S.); 2 Key Laboratory of Wildlife Biotechnology and Conservation and Utilization of Zhejiang Province, Zhejiang Normal University, Jinhua 321004, China

**Keywords:** bayberry pomace, insoluble dietary fiber, soluble dietary fiber, physicochemical properties, functional properties

## Abstract

Bayberry pomace, a nutrient-rich material abundant in dietary fiber (DF), has historically been underutilized due to a lack of thorough research. This study aimed to investigate the physicochemical and functional properties of the DF. Ultrasonic enzymatic treatment was performed to extract the total DF, which was then optimized to produce modified soluble dietary fiber (MSDF) and insoluble dietary fiber (MIDF). The optimized conditions yielded 15.14% of MSDF with a water-holding capacity (WHC) of 54.13 g/g. The DFs were evaluated for their structural, physicochemical, and functional properties. The MSDF showed a higher (*p* < 0.05) WHC, oil-holding capacity (OHC), swelling capacity (SC), cation exchange capacity (CEC), and glucose adsorption capacity (GAC) (about 14.15, 0.88, 1.23, 1.22, and 0.34 times) compared to the DF. Additionally, the MSDF showed strong, superior radical scavenging and blood sugar-lowering capabilities, with a more porous surface morphology. A Fourier-transform infrared (FT-IR) spectroscopy analysis indicated that enzymatic modification degraded the cellulose and hemicellulose, reducing the DF crystallinity. Overall, the results demonstrated that cellulase hydrolysis could effectively improve the physicochemical and functional properties of DF, thereby paving the way for its development into functional food products.

## 1. Introduction

Bayberry (*Myrica rubra Sieb. et Zucc.*) is distributed in the middle and lower reaches of the Yangtze River in China, where it has been cultivated for centuries. Zhejiang is one of the primary regions for bayberry production, and its cultivation and yield rank the highest in the country [1]. Bayberry fruit is abundant in proteins, vitamins, citric acid, polysaccharides, polyphenols, and other essential nutrients [2,3,4]. Bayberry extracts have been used in China as astringents and antidotes or for the treatment of diarrhea, digestive problems, headache, burns, and skin diseases [2]. However, bayberry matures during the hot and rainy season, from June to July, rendering it susceptible to spoilage and resulting in a short shelf life. Processing is a crucial method for extending its shelf life, allowing for prolonged consumption and meeting market demand. However, it also generates a significant amount of fruit residue waste [5]. The pomace generated from the processing of bayberry wine, juice, and other products is often used as animal feed or discarded outright, resulting in a loss of valuable nutrients, including sugars, polyphenols, and dietary fiber [6,7,8,9]. Therefore, it is necessary to enhance the research on the nutritional and bioactive compounds present in bayberry pomace, aiming to mitigate the environmental impact and facilitate its application in the food industry.

Of these compounds, dietary fiber (DF) is a macromolecular polysaccharide containing numerous active groups such as amines, carboxyls, hydroxyls, and ketone, conferring traits such as water-holding capacity (WHC), oil-holding capacity (OHC), adsorption, and reversible exchange properties [10] and thus imparting it with significant nutritional and medical value [11]. DF is generally classified as water-soluble dietary fiber (SDF) or water-insoluble dietary fiber (IDF), with natural foods containing mostly IDF (about 70–80%) [12]. The application of high-pressure homogenization significantly augmented the SDF content in DF, concurrently enhancing its physicochemical properties and biological functionalities [13]. Animal studies have revealed that DF demonstrates several biological activities. For instance, IDF shows a beneficial capacity for adsorbing toxic and harmful substances within the intestinal tract, thereby lowering the risk of colon cancer and alleviating constipation [14]. SDF has a better WHC and higher viscosity, which can better reduce the postprandial blood glucose level and cholesterol adsorption capacity. These effects are determined by the source of dietary fiber, which may affect the chemical structures and compositions [6]. Moreover, the poor smell and taste of plant-derived DF have affected its direct application in food. Hence, it is crucial to find effective methods to modify DF [15]. At present, physical, chemical, or biological methods, or multiple methods, may be used to improve the physicochemical and functional properties of DF [16,17,18]. Ultrasonic enzyme treatment is widely applied in the modification of DF due to its safety, minimal impact on the molecular structure of DF, and enhanced solubility and effectiveness of SDF content [19].

However, there has been limited research on the preparation or properties of bayberry pomace DF. In order to utilize bayberry pomace and explore the potential application of bayberry DF in functional foods, DF was extracted using an ultrasound-assisted enzyme and the modification conditions were optimized, using the orthogonal method to study the effects of the fluid–material ratio, cellulase dosage, and hydrolysis time on the yield of water-soluble dietary fiber (SDF), along with its water-holding capacity (WHC). Then, the structural, physicochemical, and functional properties of the dietary fiber were evaluated and compared, so as to provide research data and a theoretical basis for its application in foods.

## 2. Results and Discussion

### 2.1. The Modification of DF from Bayberry Pomace

#### 2.1.1. Effect of Fluid–Material Ratio on the MSDF Yield and WHC

**Figure 1:**
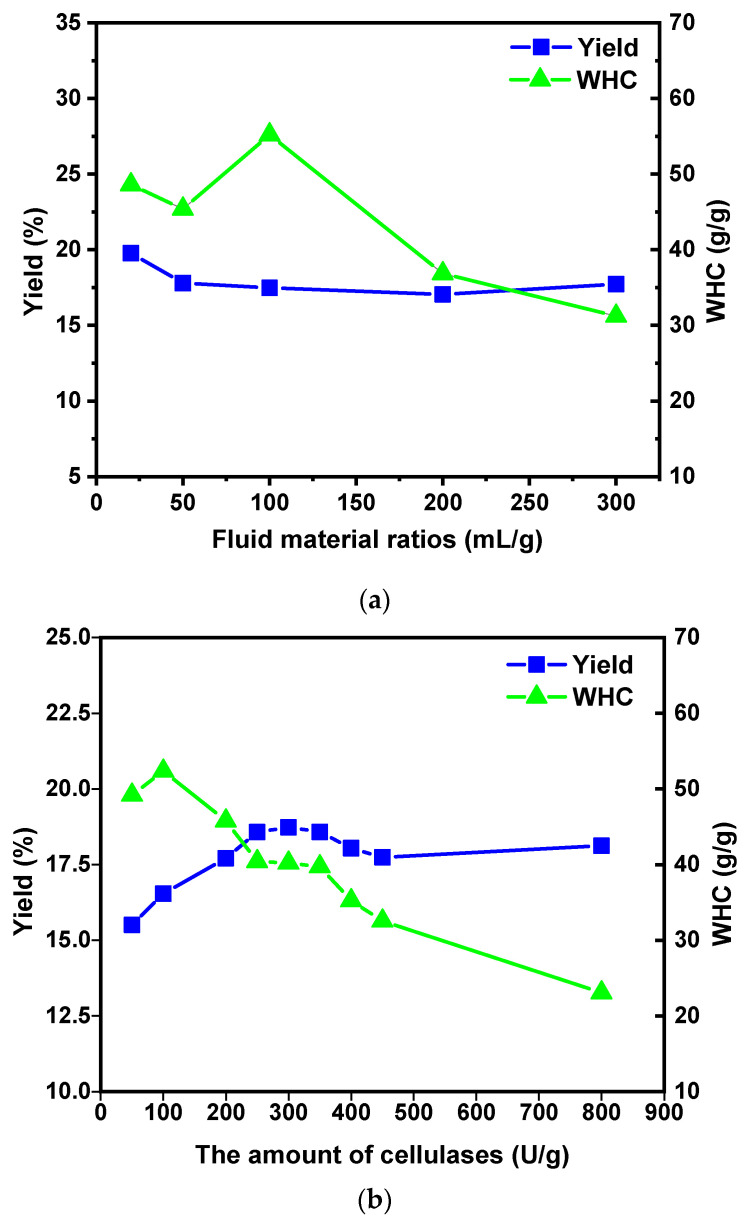

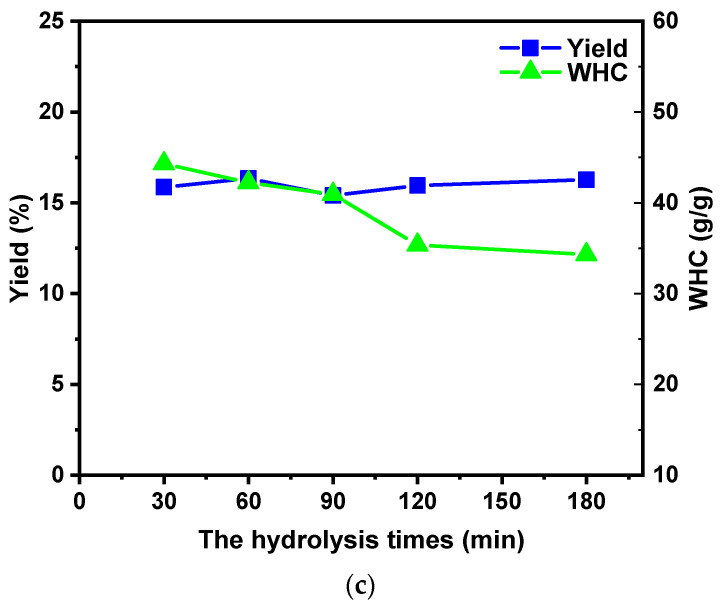
Effects of different fluid–material ratios (**a**), cellulase dosages (**b**), and hydrolysis times (**c**) on MSDF yield and WHC.

The quantity of the cellulase enzyme used was 100 U/g, and the hydrolysis time was 60 min. The effect of different fluid–material ratios on the MSDF yield is shown in Figure 1a. The yield of the MSDF decreased with the increase in the fluid–material ratio, and the WHC rose first and then decreased. When the fluid–material ratio was 20 mL/g, the highest yield of the MSDF was 19.77%. When it was 100 mL/g, the MSDF had the highest WHC, at about 55.19 g/g. It was reported that the fluid–material ratio determined the material concentration in the enzymatic reaction [11]. With an increase in the ratio, the hydrolysis degree of the MSDF molecules increased, resulting in a decrease in the yield. At the same time, the enzyme treatment could reduce the particle size of the DF, increase the structural loose pores, and thus expose more binding sites to improve the WHC. However, excessive hydrolysis led to the hydrolysis of the MSDF to smaller molecular polysaccharides, and the WHC decreased continuously [6].

#### 2.1.2. Effect of Cellulase Dosage on the MSDF Yield and WHC

As shown in Figure 1b, the MSDF yield and WHC increased and then decreased with an increase in the cellulase dosage. When the fluid–material ratio was 20 mL/g and the hydrolysis time was 60 min, the best yield was 18.73% at 300 U/g, while the highest WHC at 100 U/g was 52.36 g/g. Thus, it can be seen that a large amount of enzyme would excessively hydrolyze IDF and MSDF to glucose, affecting the MSDF yield. At the same time, MSDF would lose numerous hydrophilic groups when undergoing excessive hydrolysis, resulting in the continuous decrease in the WHC [20].

#### 2.1.3. Effect of Hydrolysis Time on the MSDF Yield and WHC

To investigate the effect of the hydrolysis time on the MSDF yield and WHC, the fluid–material ratio and cellulase dosage were set as 20 mL/g and 100 U/g, respectively. The results are shown in Figure 1c. The WHC exhibited an inverse relationship with the hydrolysis time. The highest WHC, at 44.31 g/g, was observed at 30 min and thereafter decreased steadily with a prolonged reaction time. On the other hand, the MSDF yield showed a slight increase followed by a slight decrease, peaking at approximately 16.34% after 60 min. When the substrate, cellulase dosage, and reaction condition were constant, some of the IDF in the DF hydrolyzed to MSDF. As the reaction progressed, the MSDF accumulated steadily. Then, the cellulase bound to the MSDF increased until reaching equilibrium and subsequently declined [20]. However, the WHC decreased continuously, which was due to the hydrolysis of the MSDF to small-molecule polysaccharides, and a large number of hydrophilic groups were lost due to enzymatic digestion.

### 2.2. Orthogonal Test to Optimize the Modification Process

The MSDF yield and WHC were used as the investigation indicators, and an L9(3^4^) orthogonal experimental design was conducted to optimize the extraction process based on the results of single-factor experiments. The experimental factors and level design are shown in Table 1, and the results are shown in Table 2.

According to the three-factor variance analysis, the fluid–material ratio, cellulase dosage, and hydrolysis time all had a significant influence on the WHC of the MSDF in the order of the hydrolysis time > fluid–material ratio > cellulase dosage. However, there was no significant effect on the yield rate of the MSDF compared with the single-factor test. Therefore, the optimal modification scheme was the A2B2C2: fluid ratio (100:1 mL/g), cellulase dosage (100 U/g substrate), and hydrolysis time (60 min). With the best modified conditions, the MSDF was prepared in triplicate, and the mean yield was 15.14% and the WHC was 54.13 g/g, which was basically in line with the expected target.

### 2.3. Physicochemical Properties

The results for the physicochemical properties, including the WHC, OHC, SC, GAC, and CEC, are shown in Table 3. The physicochemical properties of the DF showed a significant difference (*p* < 0.05). However, the properties of the MSDF and MIDF (WHC, OHC, SC, CEC, and GAC) were 14.15 and 1.50 times, 0.88 and 1.13 times, 1.23 and 1.07 times, 1.22 and 1.30 times, and 0.34 and 0.66 times higher than those of the DF, respectively. Obviously, except for the GAC, the physicochemical properties were all significantly improved after the DF modification. Interestingly, compared with the previous research on DF, such as *Forsythia suspensa* and *Rubus chingii*, our MSDF had a superior WHC [21]. As reported in the literature [22], the WHC was generally related to the chemical properties and number of hydrophilic points, as well as the surface area, density, and structure. The WHC of the MSDF changed, which may be related to the effect of cellulase on both IDF and MSDF. The digestion reaction transformed some IDF into MSDF, and some active groups, pore structures, and cross-link structures in the MSDF were destroyed, leading to a decrease in the water-holding performance. Meanwhile, excessive enzymatic digestion will also lead to a small particle size for IDF and affect its functional properties. As a hydration ability, the SC depends on different factors, such as the chemical structure, processing parameters, porosity, intermolecular association, and so on [21]. After the modification, the WHC of the MSDF and MIDF increased, with a loose structure, multilayer folds, and larger pores, and the specific surface area increased, meaning that more hydrophilic groups such as hydroxyl and carboxyl groups were exposed to increase the binding site of the water. However, the OHCs of the DF and MIDF were higher than that of the MSDF, which may be related to the lower lignin content of MSDF. The situation for the CEC was that the MSDF and MIDF were stronger than the DF due to the moderate crushing and exposure of the partial uronic acid group after modification. However, there were some discrepancies compared to the research of Wang [21] and He [20].

### 2.4. The Radical Scavenging Capacity 

**Figure 2:**
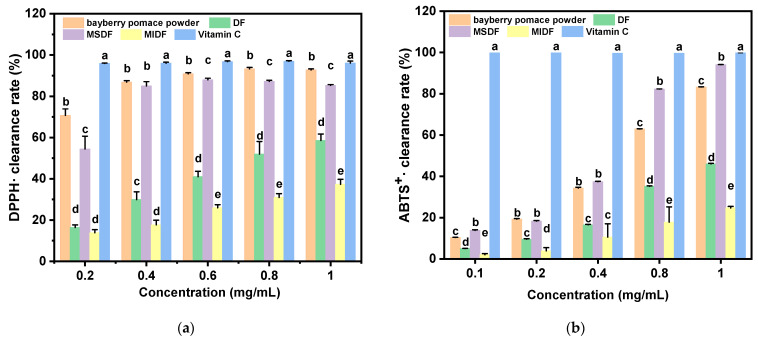
The radical scavenging activity of DF, MIDF, MSDF, and DP. (**a**) The DPPH· radical scavenging activity; (**b**) the ABTS^+^· radical scavenging activity. Different letters (a, b, c, d, and e) refer to significantly different means at *p* < 0.05 (Duncan’s test).

The effects of the radical scavenging capacity of DF before and after modification are shown in Figure 2. From the data in Figure 2a, it can be observed that the DPPH· radical scavenging activity was positively correlated with each dietary fiber sample concentration. In particular, the bayberry pomace powder and MSDF showed a higher scavenging activity on the DPPH· radicals. In addition, each concentration of the MSDF group showed a significant difference in comparison to the DF and MIDF groups (*p* < 0.05), which was consistent with the report on black mulberry [23,24]. Notably, the best scavenging activity of the MSDF against DPPH· radicals was 87.95 ± 0.79% at a concentration of 0.6 mg/mL. The scavenging capacity of the DPPH· radicals was shown to be DP > MSDF > DF > MIDF after the modified treatment. However, the scavenging capacities were slightly lower than those of the positive control group (Vitamin C).

The results of the evaluation of the ABTS^+^· radical scavenging activity are presented in Figure 2b. All the types of dietary fiber samples exhibited ABTS^+^· radical scavenging activity with a positive dose-dependent manner. There were exceptions for the concentrations of 0.2 and 0.4 mg/mL, but the MSDF under other concentrations showed a significant difference (*p* < 0.05) in comparison to the DF, MIDF, and DP. When the dose of MSDF reached 1.0 mg/mL, the scavenging activity of the ABTS^+^· radical was 94.05 ± 0.10%, which was clearly comparable to Vitamin C. Likewise, the scavenging capacity of the ABTS^+^· radical was MSDF > DP > DF > MIDF after the modification.

The MSDF exhibited strong radical scavenging abilities against the DPPH· and ABTS^+^· radicals, consistent with the findings of Afrazeh [25]. This radical scavenging potential of the dietary fibers may be attributed to the free carboxyl groups and hydroxyl groups, as well as to some polyphenols. In the study of SDF and IDF from quinoa and wheat, Chen et al. found that the radical scavenging activity of SDF was significantly stronger than that of IDF, which was probably due to its higher content of uronic acid and the presence of hydroxyl groups in the main backbone [26]. In an early study, Hu et al. evaluated the radical scavenging activities of highly purified monosaccharides, oligosaccharides, and complex carbohydrates and revealed that the radical scavenging capacity of MSDF may be related to its glycosidic bonding and molecular chain-binding substances, such as phenolic and/or protein components [27]. In addition, it was reported that bayberry pomace, as a processing by-product, is rich in phenols [1,28]. The hydroxyl groups of phenolic compounds could donate electrons or hydrogen atoms to enhance the radical scavenging activity [6,29].

### 2.5. Hypoglycemic Capacity In Vitro

**Figure 3:**
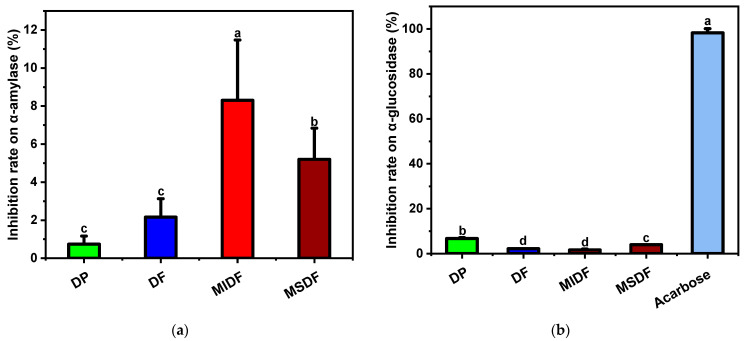
The hypoglycemic effects of DP, DF, MIDF, and MSDF. (**a**) The inhibition of α-amylase with 1.0 mg/mL of samples. (**b**) The inhibition of α-glucosidase with 10 mg/mL of samples. Different letters (a, b, c, and d) refer to significantly different means at *p* < 0.05 (Duncan’s test).

Generally, dietary starch is processed by α-amylase into maltose and dextrin, which might then be changed by α-glucosidase into glucose, increasing the blood glucose level [30,31]; therefore, the inhibition rates of α-amylase and α-glucosidase can indirectly aid in the judgment of the hypoglycemic capacity in vitro. In this study, the hypoglycemic effects of DP, DF, MIDF, and MSDF were studied based on the inhibition of α-amylase and α-glucosidase, and the results are shown in Figure 3. There were significant differences between the MIDF and MSDF groups compared to the DF group, as was similar to the previous findings [14]. The effect of each of the dietary fibers on α-amylase inhibition is shown in Figure 3a; the inhibition rates of the MIDF and MSDF (1.0 mg/mL) were 8.3% and 5.2%, which was 11.26 and 7.03 times higher than that of the DF, respectively. The inhibition rates of α-amylase were roughly in the order of MIDF > MSDF > DF > DP. These results are probably related to the exposure of the internal structure and polar groups of MIDF and the improvement in the physical barrier [14]. Contrarily, the inhibitory activity of the MSDF was 62.65% of that of the MIDF, perhaps because of the looser structure and the higher WHC, which decreased the contact rate between the α-amylase and starch [14,32]. 

The inhibition rate of α-glucosidase is shown in Figure 3b. It was found that each dietary fiber of bayberry pomace had a limited ability to inhibit the catalytic behavior of α-glucosidase before and after modification. In particular, there was no significant difference between the DF and MIDF. The inhibitory effect of the MSDF was 3.99%, compared to the standard drug, which was about 4.06% of the acarbose. Previous studies reported that SDFs limited the α-glucosidase when binding with the substrate for the viscosity effect at a high concentration [33,34]. However, another study determined that six SDFs did not significantly hinder the α-glucosidase, indicating that the presence of SDFs did not interfere with the contact of the enzyme with the substrate [35]. It was further confirmed that the binding sites of an enzyme or the enzyme–substrate complex are affected comprehensively by the SDFs [35,36]. In this study, we conducted experiments under the substrate concentration of 10 mg/mL (1%), which was higher than the 0.004% reported before [35]. It is still worth noting that the structures of MIDF and MSDF should be explored in detail.

### 2.6. SEM Analysis 

**Figure 4:**
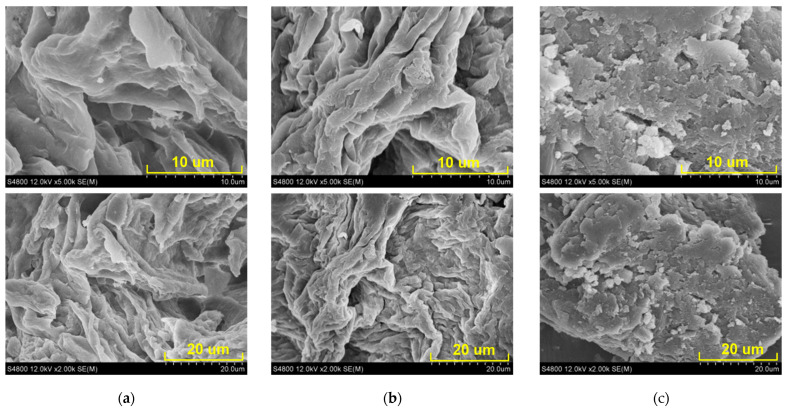
Scanning electron microscopy (SEM) of different dietary fibers from bayberry pomace before and after enzymatic modification, magnified ×5000 (5.0 k) and ×2000 (2.0 k) with scale bars of 10 µm and 20 µm, respectively. (**a**) Original DF; (**b**) MIDF; and (**c**) MSDF.

A common method was used to investigate the dietary fibers: the SEM micrographs of the DF, MIDF, and MSDF before and after modification were magnified ×2000 and ×5000. As shown in Figure 4, the SEM images clearly indicated the significant variations in the surface topography between the DF, MIDF, and MSDF. In Figure 4a, the DF shows a compact and integrated texture with obvious wrinkles, which might be from the vascular tissue of the bayberry, which supports plant growth [14]. A smooth and uniform surface with a small amount of starch granules was also visible, indicating that the structure of the DF was not destroyed during the extraction. The main structure of the MIDF was similar to the DF, but the surface was rough, and more gap cracks appeared (Figure 4b) due to the degradation of the enzyme. Previous studies found that alkaline hydrogen peroxide treatment could decrease biomass recalcitrance and facilitate hemicellulose degradation and delignification [37]. In this study, the IDF was treated with cellulase, which could degrade the cellulose, hemicellulose, and lignin. After the modification, the gap cracks in the MIDF that appeared were the result of cellulase degradation, and they caused an increase in the specific surface area, meaning that more hydrophilic groups such as hydroxyl and carboxyl groups became exposed, resulting in an increased WHC (Table 3). However, the structure of the MSDF (Figure 4c) was completely different to the DF and MIDF. There were multiple layer fragments, and the structure was loose and featured particles or lumps on the surface of the MSDF. The cellulase modification boosted the rupture of the MSDF, and the inner structure was largely exposed, resulting in decreased polymerization. In their studies on wheat dietary fiber, Chen et al. [26] found that wheat-soluble dietary fiber had a relatively flat and loose structure with gaps between fibers, while wheat-insoluble fiber had lots of small cracks and clumps on its surface. However, the biomass recalcitrance of SDF toward enzymatic attack was obviously weaker than that of IDF, which promoted enzyme binding to the substrate. The results obtained were similar to the results of dietary fiber in *litchi* found by Li et al. [6]. In conclusion, the physiochemical properties of dietary fiber are determined by its microstructure. Hence, a porous and folded structure can increase the specific surface area and expose more polar groups, thereby promoting the adsorption and binding of water and further affecting its applications in food [6,38].

### 2.7. FTIR Analysis 

**Figure 5:**
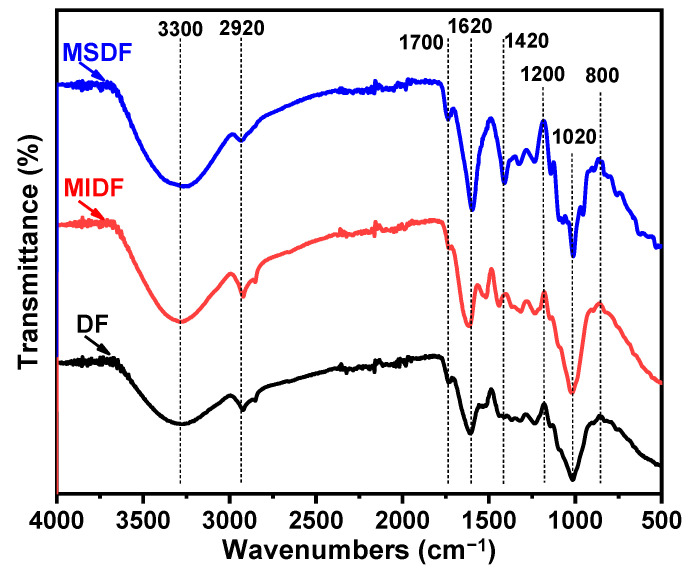
The FTIR spectra of the different dietary fibers before and after the enzymatic modification, where the blue curve corresponds to MSDF, the red one refers to MIDF, and the black one means DF.

FTIR spectroscopy is a crucial method for detecting the structural features of polysaccharide compounds, which are capable of identifying various chemical bonds and functional groups within substances [39]. The FTIR spectroscopy results of the DF, MIDF, and MSDF from 500 cm^−1^ to 4000 cm^−1^ are shown in Figure 5. The overall peak intensity of the MSDF was higher than that of the DF and MIDF, indicating that the MSDF had more typical polysaccharide complex structures [6]. There were wide absorption peaks near 3300 cm^−1^ for all of the samples, due to the stretching vibration of O–H, which mainly came from cellulose or hemicellulose [26,40]. Here, the peak intensity of the MSDF was stronger than those of the DF and MIDF, indicating more hydrogen bonds. The weak absorption band at 2920 cm^−1^ originated from the stretching of the C–H and CH_2_ groups in the substrates [41]. The absorption peaks at around 1700 cm^−1^ were due to the stretching vibration of the C=O group, while the deep peak at 1620 cm^−1^ was from the O–H group between the cellulose and water molecules [39]. The absorption peaks in the range of 1200–1420 cm^−1^ were caused by the angular vibrations of C–H, indicating the typical structure of the carbohydrate skeleton. Notably, the absorption peaks in the MSDF were significantly stronger than those in the DF and MIDF, probably due to the site exposure after the modification. The absorption peaks in the range of 1200–1000 cm^−1^ corresponded to the tensile vibrations of C–C, C–O, or C–O–C, reported as the presence of sugar aldehyde groups [42]. The absorption peaks of the β-glucoside bond and α- and β-pyranose could be seen near 800 cm^−1^. The above results indicate that the modified dietary fiber showed carbohydrate characteristics consistent with the typical polysaccharide absorption peak.

### 2.8. XRD Analysis 

**Figure 6:**
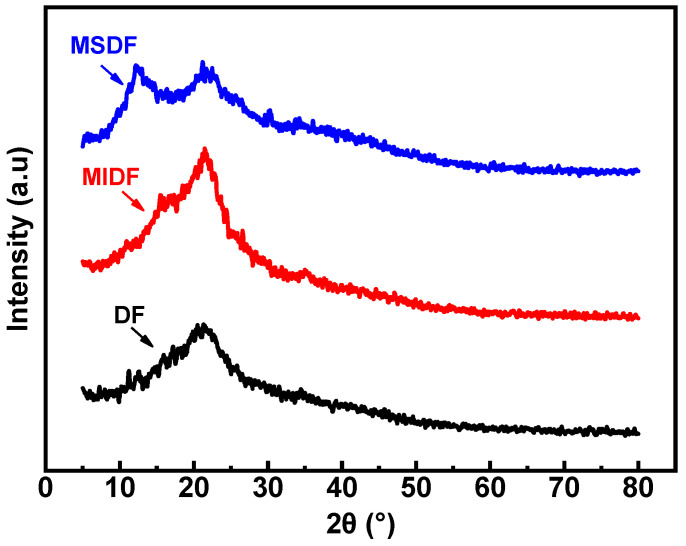
The XRD pattern of the different dietary fibers before and after the enzymatic modification, where the blue curve corresponds to MSDF, the red one refers to MIDF, and the black one means DF.

X-ray diffraction was extensively utilized to assess the alterations in crystallinity, thereby ascertaining the aggregation state of the DF molecules [43]. Based on a recent report, it was found that IDFs display crystal structures resembling cellulose type I, whereas SDFs exhibit an amorphous structure [44]. The XRD analysis depicted in Figure 6 indicates that the predominant structural characteristics of the bayberry-derived DF samples were marginally distinct, featuring broad peaks within the range of 10–30°. Specifically, the DF samples exhibited high-intensity peaks at 2θ values of approximately 11.29° and 21.45°. In contrast, the MIDF displayed a notable diffraction peak at 2θ around 22.5°, accompanied by minor diffraction peaks at 2θ values of 15.41° and 26.68°, suggesting the potential presence of crystal structures resembling cellulose type I. The diffraction patterns of the MSDF exhibited low intensity and a lack of sharp, strong peaks, suggesting difficulties in achieving complete crystallization. Specific peaks at 12.25°, 21.52°, 22.59°, and 30.29° were identified. The overall crystallinity values for the DF, MSDF, and MIDF were measured at 18.32%, 14.16%, and 7.25%, respectively. These findings indicate the potential destruction of the crystal regions in bayberry DF following enzyme modification [45]. The crystallinity value of the MIDF was marginally lower compared to the other samples and possibly attributable to the disturbance of the intermolecular connections between the cellulose microfibers, hemicelluloses, and lignin. Furthermore, the XRD pattern and crystallinity value of the MSDF closely resembled those reported in various studies, including ones examining the dietary fiber of grapefruit peel [15], sorghum [46], and *Rubus chingii* Hu. Fruits [21].

## 3. Materials and Methods

### 3.1. Materials

The bayberry fruits were harvested in May 2022 from Lanxi Fengyuan Food Technology Co., Ltd. (Lanxi, China). The raw bayberry pomace produced after juicing was stored at −20 °C for frozen preservation. The alkaline protease, α-glucosidase, α-amylase (high temperature), and cellulase were purchased from Solarbio Science & Technology Co., Ltd. (Beijing, China). The acarbose and 2,2′-azino-bis (3-ethylbenzothiazoline-6-sulfonic acid) diammonium salt (ABTS) reagent were purchased from Aladdin Biochemical Technology Co., Ltd. (Shanghai, China). The phosphate-buffer saline (PBS) and 3, 5-dinitrosalicylic acid (DNS) were prepared and used immediately. The 1,1-Diphenyl-2-picrylhydrazylradical (DPPH) and other reagents were purchased from Sinopharm Group Chemical reagent Co., Ltd. (Shanghai, China).

### 3.2. Extraction of Bayberry Pomace DF

**Figure 7:**
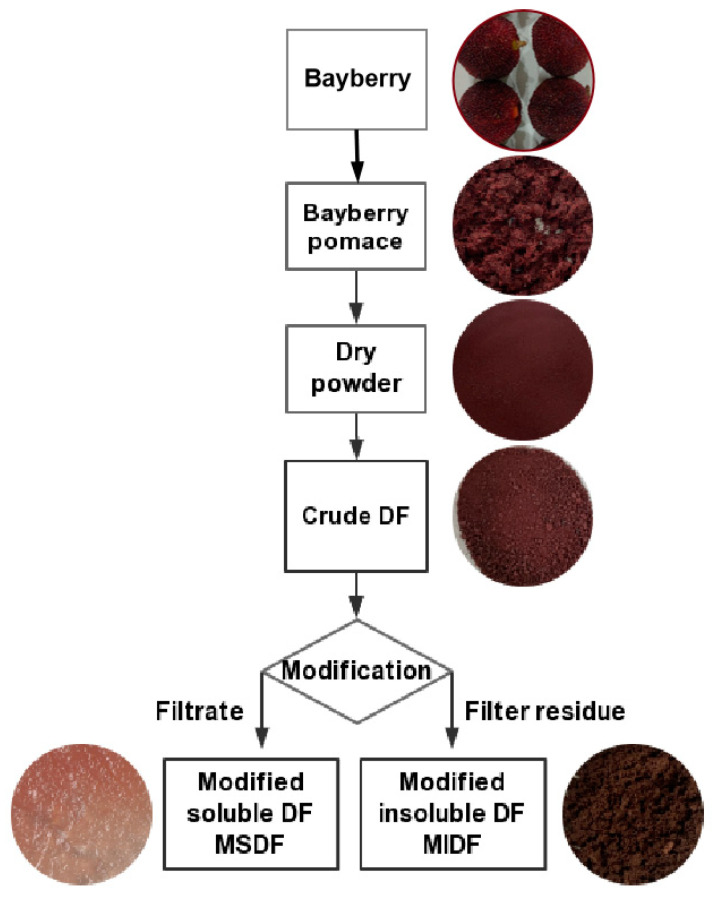
Preparation process of functional dietary fiber from bayberry pomace.

The preparation of the functional DF from bayberry pomace was divided into two main stages: extraction and modification (Figure 7). Initially, the frozen bayberry pomace was washed with deionized water twice, vacuum-dried at 60 °C for 24 h, and crushed and passed through 80 mesh to produce dry powder (DP). Then, the DP was mixed with petroleum ether (1:5, *w*:*v*) and soaked overnight at room temperature. After Buchner funnel suction filtration in a circulating water vacuum pump, the filter residue was washed with deionized water and dried at 70 °C to achieve constant weight. The fat in the DP was removed by separating the filtrate. The defatted powder was then rinsed successively with 85% ethanol solution (10 mL/g sample) three times and dried to constant weight, and some sugars in the sample were also removed with ethanol. The defatted and desugared sample was mixed with citric acid buffer solution (1:40, *w*:*v*, pH 6.0) in a conical flask, which was successively sealed with ultrasonic for 15 min (250 W, 40 kHz, and 50 °C). Then, the sample was hydrolyzed with thermostable α-amylase (10,000 U/g, expressed as enzyme activity per gram of substrate) at 95 °C for 35 min. Thereafter, alkali protease (5000 U/g) was added and incubated at pH 8.0 at 60 °C for 60 min. After hyperthermia inactivation in a boiling water bath for 10 min, glucoamylase (5000 U/g) was added and shaken at pH 4.5 at 60 °C for 30 min. Ethanol (1:4, *v*:*v*) was added to the resulting mixture, and it was incubated at 60 °C for 60 min. The DF precipitate was then obtained via suction filtration, as before, vacuum-dried at 70 °C to constant weight, and stored at −4 °C until further use. After analysis, the DF contained 8.40% SDF and 91.09% IDF.

### 3.3. Modification and Optimization of Bayberry Pomace DF

DF powder was mixed with pH 5.0 deionized water (1:50, *w*:*v*) and hydrolyzed with cellulase (*w*:*w*, 50 U/g) at 50 °C for 60 min. The mixture then underwent hyperthermia inactivation in a boiling water bath for 10 min and was centrifuged at 5000× *g* for 20 min, and then the filter residue and the filtrate were separated. (a) The residue was washed with 20 mL hot water (70 °C) and suction-filtrated, as before. The filter slag was vacuum-dried at 50 °C for 12 h to yield MIDF. (b) The filtrate was added to a 4-times quantity of ethanol, and after 60 min of sedimentation, it was centrifuged at 5000× *g* for 20 min. The MSDF was weighed before (wet weight) and after drying (dry weight). The yield of MSDF was calculated according to the following Equation (1):

(1) $$Yield\;of\;MSDF(\%)=\frac{Dried\;weight\;of\;MSDF}{total\;weight\;of\;DF}\times 100\%$$

To boost the MSDF yield, the modification conditions were optimized, considering the crucial physiological functions of SDF’s hydration properties [47]; therefore, MSDF yield and WHC were selected as the investigation objectives, and the modification process was optimized by single-factor and orthogonal tests. During this process, the fluid–material ratio (20:1, 50:1, 100:1, 200:1, and 300:1), the cellulase dosage (50, 100, 200, 400, and 800 U/g), and the hydrolysis time (0.5, 1, 1.5, 2, and 3 h) were examined.

### 3.4. Physicochemical Properties 

#### 3.4.1. WHC Analysis

Next, 1 g of DF/MIDF sample was accurately weighed and added to deionized water (10 mL) and shaken at room temperature for 60 min. Then, the precipitate was obtained after centrifugation at 5000× *g* for 20 min. The WHC was calculated after weighing, as follows [43,48].

Additionally, as soluble dietary fiber, MSDF can be completely dissolved in water. However, slight modifications were made to the WHC analysis of MSDF [43,48]: 1 g of MSDF sample was accurately weighed, added to deionized water (100 mL), and stirred for 10 min until completely dissolved in water. Before centrifugation at 5000× *g* for 40 min, the solution was added to a 4-times volume of ethanol and underwent 60 min of sedimentation. To avoid water loss, the centrifugal precipitate was instantly weighed as the weight of wet MSDF. The WHC was finally calculated as follows:

(2) $$\mathrm{WHC}\;\left(g/g\right)=\frac{M_{1}-M_{0}-m}{m},$$

where *M*_1_ is the total weight of wet DF and the centrifuge tube; *M*_0_ is the weight of the tube; and *m* is the weight of dry DF. 

#### 3.4.2. OHC Analysis

Next, 1 g of the DF/MSDF/MIDF sample was accurately weighed in a 50 mL centrifuge tube, and 10 mL of soybean oil was added; it was shaken thoroughly and maintained at 37 °C for 12 h. Then, the upper oil was discarded after centrifugation at 5000× *g* for 20 min. The OHC was calculated after weighing, as follows [43,48]:

(3) $$\mathrm{OHC}\left(g/g\right)=\frac{M_{1}-M_{0}-m}{m},$$

where *M*_1_ is the total weight of wet DF and the centrifuge tube; *M*_0_ is the weight of the tube; and *m* is the weight of dry DF.

#### 3.4.3. SC Analysis

The swelling capacity (SC) was determined using the method described by Li et al. [6]. Briefly, 1 g of the DF/MSDF/MIDF sample was accurately weighed, and the volume was measured. The sample–deionized water mixture was maintained at 37 °C for 12 h. Then, the expanded sample volume was read, and the SC was calculated as follows:

(4) $$\mathrm{SC}(\mathrm{mL}/g)=\frac{V_{1}-V_{0}}{m},$$

where *V*_1_ is the sample volume after the expansion; *V*_0_ is the sample volume before expansion; and *m* is the sample weight.

#### 3.4.4. GAC Analysis

As described in the literature [49], 1.0 g of dried DF powder (*m*) was added to a 100 mL glucose solution (100 mmol/L) in a 37 °C water bath, which was shaken for 6 h. Then, the treated sample was centrifuged at 5000× *g* for 15 min, and the supernatant was measured at 540 nm using the DNS method. The glucose adsorption capacity (GAC) was calculated as follows:

(5) $$\mathrm{GAC}(\mathrm{mmoL}/g)=\frac{n_{1}-n_{0}}{m},$$

where *n*_1_ is the moles of glucose after adsorption; *n*_0_ is the moles of glucose before adsorption; and *m* is the sample weight.

#### 3.4.5. CEC Analysis

The DF/MSDF/MIDF samples were acid-treated with HCl solution (0.10 mol/L) for 24 h and washed with deionized water until neutral with no chloride ions. Then, they were dried to a constant weight. Next, 0.1 g of the dried sample was added to 100 mL NaCl solution (50 g/L). Then, it was slowly titrated with the NaOH solution (0.01 mol/L) with phenolphthalein as an indicator, and the deionized water was used as a blank control. The cation exchange capacity (CEC) was calculated according to Formula (6).

(6) $$\mathrm{CEC}(\mathrm{mmol}/g)=\frac{(V_{1}-V_{0})\times 0.01}{m},$$

where 0.01 is the concentration of NaOH (mmol/g); *V*_1_ is the volume of NaOH (mL); *V*_0_ is the volume of NaOH as the blank control (mL); and *m* is the sample weight.

### 3.5. Radical Scavenging Activities In Vitro

The radical scavenging effect of DF was analyzed in vitro according to the literature [50,51]. To determine the DPPH radical scavenging capacities, 2 mL of sample and 2 mL of DPPH solution (dissolved in methanol, 0.1 mmol) were mixed and then allowed to stand, avoiding light reaction for 30 min. Taking a blank as a negative control, the absorbance values at 517 nm were measured. The clearance rate was calculated as follows, where *A*_0_ and *A*_s_ are the absorbance values of the blank and samples:

(7) $$DPPH\cdot clearance\;rate(\%)=\frac{A_{0}-A_{s}}{A_{0}}\times 100$$

Here, 7.4 mmol of ABTS diammonium and 2.6 mmol of potassium perbisulfite (K_2_S_2_O_8_) were mixed and left to stand for 15 h at room temperature in darkness. The mixture was again diluted with phosphate buffer (pH 7.4) to an absorbance value at 734 nm of 0.700 ± 0.002, preparing the ABTS working solution, and then 0.3 mL of sample and 3 mL of ABTS solution were mixed and the absorbance was measured at 734 nm after reaction for 6 min, where *A*_0_ and *A*_s_ are the absorbance values of the blank and samples.

(8) $${\mathrm{ABTS}}^{+}\cdot clearance\;rate(\%)=\frac{A_{0}-A_{s}}{A_{0}}\times 100$$

### 3.6. Hypoglycemic Capacities In Vitro 

#### 3.6.1. α-Amylase Inhibitory Activity Analysis (AIA) 

Here, 100 μL of DF/MSDF/MIDF sample solution and 100 μL of α-amylase solution (1 U/mL) were pre-mixed at room temperature for 15 min, and then 200 μL of starch solution (0.5%, *w*:*v*) was added and mixed well in a 37 °C water bath for 10 min. The reaction was terminated by adding 1000 μL DNS reagent. The reaction solution was heated and boiled for 5 min. After it had cooled down, 3 mL of deionized water was added to dilute, and the absorbance was measured at 540 nm with a TU-1901 spectrophotometer (PERSEE^®^, Beijing, China). The inhibition rate was calculated as follows [20]:

(9) $$\mathrm{Inhibition}\mathrm{rate}(\%)=(1-\frac{A_{s}-A_{1}}{A_{0}})\times 100\%$$

where *A*_0_, *A*_s_, and *A*_1_ are the absorbance values of the blank, samples, and negative control.

#### 3.6.2. α-Glucosidase Inhibitory Activity Analysis

Briefly, 1 mL of DF/MSDF/MIDF/acarbose sample solution (10 mg/mL) and 1 mL of PNPG substrate (10 mmol/L) were mixed with 1 mL of PBS solution (pH 6.8, 0.1mol/L). After preservation at 37 °C for 5 min, 2.5 mL of α-glucosidase solution (4 U/mL) was added and mixed well. Then, it was reacted in a 37 °C water bath for 60 min, and the reaction was terminated by the addition of 2 mL of Na_2_CO_3_ solution (0.2 mol/L). The absorbance at 410 nm was measured using a TU-1901 spectrophotometer. The α-glucosidase inhibition rate was calculated as in Equation (9).

### 3.7. SEM Analysis

The DF, MSDF, and MIDF were dried, crushed to 80 mesh (0.20 mm), and sprinkled on a conductive adhesive, which was ion-sputtered with gold for 30 s. An electron microscope (S-4800, Hitachi Ltd., Tokyo, Japan) captured the scanning images at an accelerating voltage of 12.0 kV. The micrographs were taken at ×2000 and ×5000 magnification.

### 3.8. Fourier-Transform Infrared (FT-IR) Spectroscopy Analysis 

The organic functional groups of the samples were analyzed using an FT-IR spectrophotometer (Nexus 670, Thermo Fisher Scientific Inc., Waltham, MA, USA). The samples were finely ground and crushed well. Then, they were scanned with an infrared spectrometer and a diamond ATR in a spectral range of 4000–400 cm^−1^ with a resolution of 4 cm^−1^.

### 3.9. X-ray Diffraction (XRD) Analysis 

The crystalline structures of the DF samples were analyzed by utilizing an X-ray diffractometer (Bruker D8 Advance, Berlin, Germany) equipped with a CuKα radiation source (wavelength: 0.154060 nm; voltage: 40.0 kV; and current: 40.0 mA) across a 2θ angle range of 5–80° at a scanning rate of 2°/min. 

### 3.10. Statistical Analysis 

All the measurements were performed at least in triplicate, and the results are expressed as the mean values with their standard deviations. The statistical analysis was carried out via SPSS ver. 21.0 (SPSS Inc., IBM Corporation, Chicago, IL, USA), and the data were analyzed with one-way ANOVA and Duncan’s test. *p* < 0.05 was considered to be statistically significant. The experimental data were processed and drawn using Origin 2018 software (OriginLab, Northampton, MA, USA).

## 4. Conclusions

In the present study, we prepared and modified the functional dietary fiber samples of bayberry pomace through ultrasonic enzymatic treatment. Then, the structural and functional properties were reported, which was helpful in understanding the nutritional and utilization value. The physicochemical properties were all significantly improved after DF modification, except for the GAC. The MSDF had a superior WHC and SC, while the MIDF exhibited a better OHC and CEC. In the comparison of the functional characteristics, the MSDF performed well, with the highest clearance rate of DPPH and ABTS free radicals, while the hypoglycemic capacity was lower. All the dietary fiber samples had typical polysaccharide functional groups and different morphology, and the crystallinity of the DF was decreased after the modification, indicating the potential destruction of crystal regions in bayberry DF following enzyme modification. It is worth noting that the MSDF exhibited strongly hydrophilic and radical scavenging properties, which might be well suited for use in formulated foods to reduce calories, avoid syneresis, prolong stability, and modify the texture. It is of great significance to improve the reuse potential of bayberry fruit pomace.

## Figures and Tables

**Table 1:** The combined test factors and horizontal design of the orthogonal test.

Level	Factors		
	A (Fluid–Material Ratio)	B (Cellulase Dosage)	C (Hydrolysis Time)
−1	20 mL/g	50 U/g	30 min
0	100 mL/g	100 U/g	60 min
1	200 mL/g	200 U/g	120 min

**Table 2:** Orthogonal test design and results.

Test Number	A	B	C	WHC (g/g)	MSDF Yield (%)
1	−1	−1	−1	45.53	15.36
2	−1	0	0	50.44	14.10
3	−1	1	1	30.78	18.62
4	0	−1	0	50.23	15.18
5	0	0	1	41.77	14.44
6	0	1	−1	40.41	12.78
7	1	−1	1	25.09	17.52
8	1	0	−1	41.54	16.90
9	1	1	0	32.73	13.84
k1	42.25	40.28	42.49		
k2	44.14	44.58	44.47		
k3	33.12	34.64	32.54		
R	11.02	9.94	11.92		
Factor priority	C > A > B				
Optimal condition	A2B2C2				

**Table 3:** Physicochemical properties of DF, MSDF, and MIDF.

	WHC (g/g)	OHC (g/g)	SC (mL/g)	GAC (mmol/g)	CEC (mmol/g) × 10^−1^
DF	3.68 ± 0.20 ^c^	2.21 ± 0.03 ^b^	6.13 ± 0.19 ^b^	0.89 ± 0.02 ^a^	2.35 ± 0.03 ^b^
MIDF	5.77 ± 0.02 ^b^	2.47 ± 0.07 ^a^	6.67 ± 0.12 ^b^	0.59 ± 0.25 ^ab^	2.99 ± 0.13 ^a^
MSDF	54.13 ± 0.77 ^a^	1.94 ± 0.04 ^c^	7.57 ± 0.4 ^a^	0.31 ± 0.08 ^b^	2.81 ± 0.09 ^a^

Note: Different letters (a, b, and c) in the same row indicate significantly different means at *p* < 0.05 (Duncan’s test).

## Data Availability

The data reported in this study are contained within the article. The underlying raw data are available on request from the corresponding author.
